# Study protocol: a randomized controlled trial comparing the efficacy of therapist guided internet-delivered cognitive therapy (TG-iConquerFear) with augmented treatment as usual in reducing fear of cancer recurrence in Danish colorectal cancer survivors

**DOI:** 10.1186/s12885-020-06731-6

**Published:** 2020-03-16

**Authors:** Johanne Dam Lyhne, Allan ‘ Ben’ Smith, Lisbeth Frostholm, Per Fink, Lars Henrik Jensen

**Affiliations:** 1 Department of Clinical Oncology, University Hospital of Southern Denmark, Vejle, Beriderbakken 4, 7100 Vejle, Denmark; 2 Ingham Institute for Applied Medical Research, 1 Campbell Street, Liverpool, NSW 2170 Australia; 3 Research Clinic for Functional Disorders and Psychosomatics, Aarhus University Hospital, Nørrebrogade 44, bygn. 4, 1, 8000 Aarhus C, Denmark

**Keywords:** Internet-based, Digital health, Cognitive therapy, Colorectal cancer, Fear of cancer recurrence, Anxiety, Randomized controlled trial

## Abstract

**Background:**

Cognitive therapy has been shown to reduce fear of cancer recurrence (FCR), mainly in breast cancer survivors. The accessibility of cognitive behavioural interventions could be further improved by Internet delivery, but self-guided interventions have shown limited efficacy. The aim of this study is to test the efficacy of a therapist guided internet-delivered intervention (TG-iConquerFear) vs. augmented treatment as usual (aTAU) in Danish colorectal cancer survivors.

**Methods/design:**

A population-based randomized controlled trial (RCT) comparing TG-iConquerFear with aTAU (1:1) in *n* = 246 colorectal cancer survivors who suffer from clinically significant FCR (Fear of Cancer Recurrence Inventory Short Form (FCRI-SF) ≥ 22 and semi-structured interview). Evaluation will be conducted at 2 weeks, 3 and 6 months post-treatment and between-group differences will be evaluated. Long-term effects will be evaluated after one year. Primary outcome will be post-treatment FCR (FCRI-SF). Secondary outcomes are global overall health and global quality of life (Visual Analogue Scales 0–100), bodily distress syndrome (BDS checklist), health anxiety (Whiteley-6), anxiety (SCL4-anx), depression (SCL6-dep) and sickness absence and health expenditure (register data). Explanatory outcomes include: Uncertainty in illness (Mishels uncertainty of illness scale, short form, MUIS), metacognitions (MCQ-30 negative beliefs about worry subscale), and perceived risk of cancer recurrence (Visual analogue Scale 1–100).

**Discussion:**

This RCT will provide valuable information on the clinical and cost-effectiveness of TG-iConquerFear vs. aTAU for CRC survivors with clinical FCR, as well as explanatory variables that may act as outcome moderators or mediators.

**Trial registration:**

ClinicalTrials.gov; NCT04287218, registered 25.02.2020.

https://clinicaltrials.gov/ct2/results?cond=&term=NCT04287218&cntry=&state=&city=&dist=.

## Background

Colorectal cancer (CRC) screening, early detection, and improved treatment have led to rising survival rates over the past decades. This improvement has resulted in an increasing number of long-term CRC survivors with no residual disease. Most survivors manage to establish a ‘new normal’ after finishing treatment, but some survivors experience difficulties in normal functioning and decreased quality of life (QoL) due to substantial psychological strain. Anxiety and depression e.g. are reported in 34% of CRC survivors 1–5 years post-diagnosis [1]. One of the most common concerns among cancer survivors is fear of cancer recurrence (FCR) [2], defined as “*Fear, worry or concern relating to the possibility that cancer will come back or progress* [3]”. The severity of self-reported FCR does not seem to differ much according to cancer type [4] and FCR can persist even among very long term survivors [5]. Higher FCR is associated with multiple psychological factors including (health) anxiety [6], depression [7], greater uncertainty in illness, perceived risk of recurrence and negative beliefs about worry [8]. An expert consensus on the defining features of clinical FCR suggested, that the following four features are key characteristics of clinical FCR: a) high levels of preoccupation; b) high levels of worry; c) that are persistent; and d) hypervigilance to bodily symptoms [9].

Most CRC survivors report some degree of FCR [10–13]. The term “clinically significant FCR” is introduced [14] to describe when the strain of FCR becomes clinically important, negatively influencing the life of the survivor. Validated screening questionnaires, such as the Fear of Cancer Recurrence Inventory-Short Form (FCRI-SF) [15], have been used to identify likely cases of clinically significant FCR. Two recent studies report likely clinically significant FCR based on the FCRI-SF among 13,7% [16] and 10,1% [17] of CRC survivors (unpublished data, personal communication with first authors). However, the prevalence of clinical FCR in CRC survivors is still somewhat uncertain, as estimates are based on small studies [*n* = 51–91, 11, 12, 16, 17), or studies with simplistic [10, 13] or unvalidated [12] FCR measures.

Two large cohort studies have focused on patient reported health-related QoL after (colorectal) cancer. The English study [1, 18, 19] includes people “living with and beyond cancer”, which does not distinguish between survivors with no residual disease, those living with cancer or with a history of recurrence. Furthermore, FCR is assessed with a single item. A Dutch study based on the PROFILES registry [4] used the Impact of Cancer scale (Health Worries subscale) measure, which does not include a proposed cut-off score for clinical FCR. The current study will provide a more definitive estimate of the prevalence of clinical FCR in CRC survivors.

This study will also explore psychological factors related to FCR in CRC survivors. A cancer diagnosis is life changing and imposes heavy stress on patient and relatives. Together with often numerous physical symptoms and social changes after the cancer treatment, the net sum of stressors may exceed the cancer survivor’s ability to adapt. This overload may manifest in the experience of bodily symptoms and in some cases develop into a functional disorder/somatic symptom disorder such as bodily distress syndrome or health anxiety, as proposed by Simonelli et al. [20]. Bodily distress syndrome is defined as a condition in which the patient suffers from, usually multiple, bodily symptoms in a characteristic pattern not attributable to verifiable, conventionally defined diseases [21].

Health anxiety is characterized by preoccupation with fear of having a serious and life-threatening illness with no objective sign of disease, which persists despite medical reassurance [22, 23]. Health anxiety and FCR overlap somewhat, as they both include unpleasant thoughts or ruminations, which interfere with everyday life and may lead to further unnecessary investigations and treatments. One study investigated hypochondriasis in breast cancer survivors and found that 43% of those with a clinical level of FCR met the diagnostic criteria [24]. Two studies of one CRC cohort have measured somatization (i.e. manifestation of physiological distress as physical symptoms), but not links with FCR [25, 26]. To the best of our knowledge, no previous studies have investigated the relationship between functional disorders, FCR, anxiety and depression in CRC survivors.

Illness uncertainty has been linked with FCR [27] and health anxiety [28]. When diagnosed asymptomatic through screening, illness uncertainty might by heightened. Therefore, diagnosis via screening may lead to increased issues in coping with the cancer and FCR. The comprehensive Danish Clinical Cancer Registries contain data on the method of diagnosis, namely whether the CRC survivor was diagnosed through the Danish nationwide Colorectal Cancer Screening Program, as opposed to diagnosed as a result of symptoms. This enables research in this unexplored area of psychosocial consequences of screen-detected cancers.

Around one fourth (26,5%) of CRC survivors [1] and 20–56% of people living with and beyond CRC [29] report psychosocial assistance in coping with FCR to be an important unmet need. Randomized controlled trials testing interventions for reducing FCR have primarily been conducted in breast or mixed cancer survivor populations [30]. Most interventions are based on variations of cognitive-behavioural therapy (CBT). Contemporary CBTs aiming to modify cognitive *processes* (e.g., attentional bias and beliefs about worry) rather than thought *content* (e.g. thoughts of death) were more effective (*g* = 0.42 vs 0.24). The delivery format of interventions previously or currently being evaluated has been group [31–33], face-to-face [34–36], blended [37], by telephone [38, 39] or by web-based platforms [40–42].

“ConquerFear” [34] is an individual face-to-face therapist-delivered intervention with demonstrated efficacy in reducing FCR compared to a relaxation training attention control group of patients with mixed cancers of whom the majority (89%) were women with breast cancer. While use of ConquerFear has been sustained by many study therapists beyond the end of the study [43], it is a resource and time-consuming approach accessible primarily to those in close proximity to major metropolitan cancer centres with highly trained psychologists. Consequently, a web-based self-management version of ConquerFear has been created (iConquerFear), similar in curriculum content but different in delivery. Qualitative evaluation of the usability of iConquerFear showed: iConquerFear was normalising and empowering; flexible access was key; delivery mode was engaging; tailoring was crucial; links to additional resources were valued [44].

Web-based interventions have the potential to fill an important gap in quality cancer care by augmenting limited available mental health services [45]. However, there is some evidence that entirely self-guided web-based FCR interventions may have limited efficacy, and it has been suggested that therapist input may increase efficacy [46]. Web-based therapist-guided cognitive therapy has advantages for both patients and providers and effects appear comparable to traditional face-to-face therapy in treating distress in patients with cancer [47, 48]. Evidence suggests that guided web-based interventions are superior to unguided interventions [49].

## Methods/design

### Aim

The primary aim of this RCT is to test if a therapist-guided version of iConquerFear (TG-iConquerFear) can reduce FCR and improve QoL for CRC survivors more than augmented treatment as usual (aTAU).

Secondary objectives are to

i) outline the prevalence of FCR in a population based CRC cohort up to 5 years post-diagnosis using a validated FCR measure with a clinical cut-off. This comprehensive screening will also be used to recruit to the RCT of TG-iConquerFear.
ii) outline the prevalence of anxiety, depression, bodily distress syndrome and health anxiety in a population based CRC cohort up to 5 years post-diagnosis.
iii) investigate whether being diagnosed as a consequence of the Danish Nation-wide Colorectal Cancer Screening Program increases FCR compared to being diagnosed based on physical symptoms and whether this relationship is mediated by increased uncertainty in illness.
iv) investigate whether FCR is associated with anxiety, depression, bodily distress syndrome and health anxiety in CRC survivors, as well as investigate whether uncertainty in illness, negative beliefs about worry and perceived risk of cancer recurrence act as moderators or mediators of these relationships.
v) examine the cost-effectiveness of the TG-iConquerFear intervention versus aTAU.

### Design

#### Survey

The study is population based and cross sectional. Participants will be invited to complete an electronic questionnaire to screen for FCR and other psychological factors.

#### RCT

This part of the study is a population based, randomized, controlled clinical superiority trial. Participants are randomized to internet-based, TG-iConquerFear or aTAU (1:1). All participants will follow the standard cancer follow-up program.

#### Setting

The prioritization of psychosocial aspects of cancer survivorship is derived from patient and public involvement via the Patient and Relatives Council at Lillebaelt Hospital and has led to the Clinic of Long-term Adverse Effects hosted by the Department of Oncology from where this project arises.

Experienced therapists working at the Center for Functional Disorders at Aarhus University Hospital will facilitate TG-iConquerFear. Sufficient introduction and training in iConquerFear will be ensured and supervision will be offered throughout the study. The principal investigator will be trained to perform semi-structured diagnostic telephone interviews with study participants.

The self-directed version of iConquerFear was developed, tested and refined in Australia [44]. It will be translated and adapted to a Danish context inspired by the cross-cultural adaptation process proposed by Beaton et al. [50] with random samples translated forward-backward. An expert panel of therapists with expertise in internet-based treatment will perform further testing and refinement of the intervention. A panel of volunteer, independent users from the Patient and Relatives Council will be invited to comment on all aspects of the study, including the website and written material, and continue as an advisory board throughout the project. Once complete, a pilot study of TG-iConquerFear will be conducted, see part 3.

### Study population

All Danish CRC survivors above the age of 18 diagnosed after implementation of the CRC screening programme in March 2014 who have completed curative intent cancer treatment with surgery and/or radiation and/or adjuvant chemotherapy will be invited to participate in the survey. The incidence of CRC in Denmark is 5000/year, 80% of patients are offered curatively intended treatment and the rates of 1-year and 5-year survival (data from 2011 to 2015) are 85 and 65% respectively. Assuming an overall survival rate of 75% for those offered curatively intended treatment the estimated number of invitees is 15,000.

**Fig. 1:**
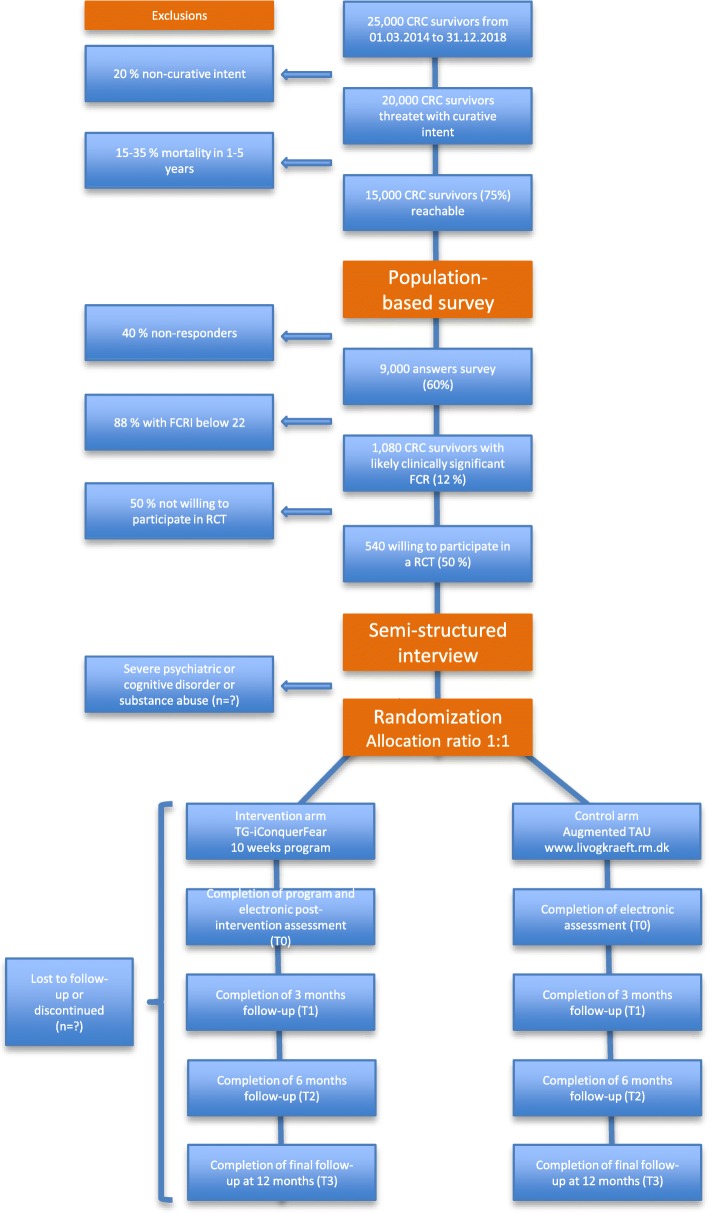
Study Timeline

RCT participants will be recruited among the 15,000 colorectal cancer survivors invited to take part in the survey. Through cooperation with the Danish Cancer Society a slightly higher response rate than previously reported [51] is expected of 60%, i.e. 9000 responders. Among these, 12% (1080) are expected to score 22 or above on the Fear of Cancer Recurrence Inventory – Short Form (FCRI-SF) [16]. Approximately half of which (*n* = 540) we expect will be willing to participate in a randomised trial. See Fig. 1 for elaboration.

Survivors will be identified through the Danish Colorectal Cancer Group (DCCG) database hosted by The Danish Clinical Registries (RKKP) with no need for the researchers to access individual patient records. The registry contains baseline data on age, sex, cancer type and TNM stage, date of diagnosis, information about performed surgery, performance status and if the patient was identified through the CRC screening program (“Yes” is annotated for individuals who submit a stool sample within three months after receiving screening invitation).

### Inclusion criteria for participating in the RCT

- Completed curative intent colorectal cancer treatment with surgery and/or radiation and/or adjuvant chemotherapy between 01.03.14 and 31.12.18
- No history of recurrence after primary operation
- Fear of Cancer Recurrence Inventory score of 22 or above [14]
- Age 18 or above
- Reads and understands Danish
- Access and ability to use Internet

### Exclusion criteria for participating in the RCT

- Cancer recurrence at any follow-up
- Inability to comply with the protocol due to severe psychiatric, cognitive disorder or substance abuse identified during telephone interview
- As the intervention is web-based participants without knowledge of or access to the Internet will be excluded from the RCT (including dyslexia).

### Procedure

#### Part 1, screening

The CRC survivors will be invited by email to their secure, personal electronic inbox to electronically complete the 61-item screening questionnaire comprising: The FCRI-SF assessing FCR severity, bodily distress syndrome (BDS checklist), health anxiety (Whiteley-6), anxiety (SCL4-anx), depression (SCL6-dep) and two items assessing global health and QoL (Visual Analogue Scales (VAS) 0–100). Two items ask for time since last cancer surveillance visit at the hospital and time until next visit. Participants are asked to answer yes or no to having received chemotherapy and/or radiation. One item asks for consent to answers being used for research in confidentiality.

Completed questionnaires will be exported directly into the REDCap database for clinical research. Those with no access to the Internet will be invited by paper and the data will be manually entered in REDCap by an investigator. In case of no response, a reminder will be sent after 2 weeks. All participants will receive feedback regarding their current level of FCR based on their answers.

#### Part 2, recruitment

In cases where participants in Part 1 report an FCRI-SF score ≥ 22 indicating likely clinically significant FCR [14], the system will automatically ask if they want to hear more about a psychological study targeting FCR, and 12 more questions will appear on illness uncertainty, negative beliefs about worry, and perceived risk of cancer recurrence along with the full version of the FCRI. See Table 1 for participant timeline [52]. If they do not want to participate further, they will be given the opportunity to state why.

**Table 1:** Participant timeline

	STUDY PERIOD					
	Population Screening	Enrolment	Allocation	Intervention	Follow-up	Close-out
**TIMEPOINT**	***-t***_***2***_	***-t***_***1***_	***t***_***b***_	***t***_***i***_	**t**_**0**_**, t**_**1**_**, t**_**2**_	***T***_***3***_
**ENROLMENT:**						
**Screening**	X					
**Semi-structured interview**		X				
**Informed consent**	X	X				
**Allocation**			X			
**INTERVENTIONS:**						
***TG-iConquerFear***				X		
***aTAU***				X		
**ASSESSMENTS:**						
***FCRI-SF***	X			X		
***Perceived risk of recurrence***	X					
***Supplementary***	X					
***Full FCRI***		X			X	X
***SCLs***	X				X	X
***BDS***	X				X	X
***Whiteley-6***	X				X	X
***QoL***	X				X	X
**Mediators:**						
***MUIS***		X		X	X	X
***MCQ-30***		X		X	X	X
***Perceived risk***		X		X	X	X

All survivors with likely clinically significant FCR who agree to be contacted by a research assistant (principal investigator or one of the two psychologists who will perform the intervention (all sufficiently trained)) will be informed by telephone about the RCT and interviewed using a modified mini-SCAN, a brief semi-structured interview for psychiatric diagnosis [53, 54]. The interview is designed to ensure appropriate treatment is offered to prospective study participants. If information and mini-SCAN cannot be delivered at the same time, a new appointment will be made. In cases of severe depression or severe psychiatric or cognitive disorder, the survivor will be excluded from the study and encouraged to seek relevant help through their general practitioner. When needed, the research assistant can consult a psychiatrist. Regular supervision will be provided.

Potential participants meeting eligibility criteria who are interested in the intervention study will receive a participant information sheet and consent form in their secure, personal electronic inbox (e-Boks). As e-Boks is accessed by NemID (a Danish secure personal coding system for electronic transactions) they will consent to participation by answering “yes”, and a concealed, computerized (REDCap) variable block randomization stratified by age and gender will take place.

#### Part 2 b, randomization

##### Control arm, augmented TAU

The control group is described as “augmented”, since the diagnostic telephone interview exceeds standard treatment. Further more, if randomized to aTAU the information letter send to their personal inbox will include a reference to a website with a non-guided, publicly available E-learning program in cancer rehabilitation hosted by the Region of Central Jutland (livogkraeft.rm.dk). In addition to written material the website includes self-help instructions for meditation. Website use will not be monitored.

##### Intervention arm, TG-iConquerFear

The information letter will contain a link to enter the platform directly. The assigned therapist will be informed simultaneously.

Both groups will follow standard surveillance according to national guidelines for cancer recurrence at a department of surgery or oncology, which may include colonoscopy and/or CT scans. Each municipality is responsible for rehabilitation and offers limited access to physical exercise, yoga, dieticians, psychologists, sexologists etc.

#### Part 3, pilot study

The first 10 invited CRC survivors allocated to TG-iCF will be evaluated separately as a pilot group to explore barriers and facilitators for entering and completing internet-based therapy. The feasibility of TG-iConquerFear will thus be optimized in a small population before launching the main RCT. To ensure continuity between screening, recruitment, and the RCT no further screening questionnaires will be sent out during pilot study evaluation. If the pilot study does indicate a need for major changes in the study design or procedures, the pilot study will seamlessly proceed to the RCT.

#### Part 4, intervention

The theoretical frame of iConquerFear [34] is based on the Common-Sense Model (CSM) of illness, the Self-Regulatory Executive Function model (S-REF; targets metacognitions) and Relational Frame Theory (RFT; theoretical basis for Acceptance and Commitment Therapy). The intervention includes elements of attention training, increasing metacognitive awareness, acceptance & mindfulness, promotion of appropriate screening behavior, and values-based goal setting. The electronic platform is accessed through secure log-on and comprises 5 modules containing educational text, interactive exercises, short videos featuring doctors, therapists and patients’ perspectives, see Table 2 and ref. for further details [44].

**Table 2:** iConquerFear curriculum content

Module	Content and Features
1. Introduction and orientation	• Introduction to FCR (survivor videos) • Overview of FCR treatment model (interactive animation and therapist video) • Values clarification (interactive card sort exercise)
2. Attention training	• Introduction to Attention Training (Interactive, therapist video, Attention Training audio, monitoring and feedback, regular practice reminders)
3. Detached mindfulness	• Introduction to Detached Mindfulness (Therapist videos) • Demonstration of Detached Mindfulness exercises (Animated videos, therapist feedback)
4. Learning to live well and manage worry	• Psycho-education regarding appropriate threat monitoring behaviours (Annotated PowerPoint video) • Assessing compliance with follow-up & self-examination recommendations (Interactive exercise with personalised feedback) • Worry management techniques (Textual overview and downloadable PDF)
5. Treatment summery and relapse prevention	• Assessment and feedback on change in FCR symptoms during treatment (therapist feedback) • Consolidation of newly acquired strategies for managing FCR through relapse prevention (therapist feedback and downloadable action plan)

Features common across all modules include: Write to your therapist, interactive exercises, downloadable hand-outs, progress graphs and safety plan

The Australian version of iConquerFear is completely self-directed. The Danish TG-iConquerFear will contain a messenger-function on the intervention dashboard allowing the participant and therapist to communicate asynchronously. The participant is guided through the sessions by minimum weekly contact with an experienced therapist (estimated ½ hour/week in 10 weeks). The therapist will motivate, answer questions and give feedback on written material and exercises. The amount of contact will be recorded and explored as a moderator of intervention efficacy. Should any cancer-related questions occur, an oncologist or surgeon will be available to consult with therapists.

Participants are asked to fill out the FCRI-SF at first log-in. Proposed mediators will be evaluated twice during the intervention. The programme can monitor adherence, dropouts and track log-ins and activity. A purpose-developed single-item questionnaire within the iConquerFear will each week ask for level of engagement [55]. Participants who do not adhere to, or withdraw from, the intervention will be contacted by telephone and asked why. These participants will be asked if they would be willing to complete a post-intervention assessment and all follow-up assessments to aid intervention evaluation. Therapist assistance is scheduled to last 10 weeks, but the participants will have free access to the platform for another 6 months.

In case of recurrence during the 10 weeks of intervention, the participant will be excluded from analysis, but may continue in the programme.

### Evaluation

#### Primary outcome measure

- Total FCRI score at 2nd follow-up 3 months post-intervention (T1) (recently validated Danish version of the FCRI) [16].
- The FCRI is a 42-item self-report measure that includes subscales assessing FCR triggers, psychological distress, severity, functioning impairments, insight, reassurance, and coping strategies [15]. The FCRI has demonstrated high interclass correlation (0.84) [16], internal consistency (α = 0.96) [56] and convergent validity in large heterogeneous cancer survivor samples. Total FCRI score will be used as the dependent variable, as it closely reflects proposed features of clinically significant FCR; namely functional impact, related distress, and maladaptive coping [3], rather than the level of fear indicated by the FCRI-SF [57, 58]. Respondents use a Likert scale ranging from 0 (‘not at all’ or ‘never’) to 4 (‘a great deal’ or ‘all the time’) to rate the degree to which symptoms and/or issues affected them over the past month. Total scores can range from 0 to 168. Higher scores indicate greater FCR morbidity. Level of FCR will be measured post-intervention (T0) and at follow-up after 3 (T1) and 6 (T2) months. Long-term effect will be evaluated after one year (T3). All electronic questionnaires will be sent out automatically through REDCap.

#### Secondary outcome measures

Change from baseline (Tb) to post-intervention (T0, T1, T2, T3) in the following outcomes

- *Bodily Distress Syndrome/somatization* evaluated by the BDS Checklist validated by Budtz-Lilly et al. [59].
- *Anxiety and depression* evaluated by the relevant Symptom Checklist-90-R (SCL) subscales [60].
- *Health Anxiety* measured by the validated Whiteley-6 index [61, 62].
- *Global quality of life* and *global health* measured by a VAS 0–10

### Process measures

Variables likely to mediate the impact of TG-iConquerFear according to the cognitive processing and blended theoretical models of FCR and the existing research will be measured twice during the intervention and at follow-up

- *Uncertainty in illness* measured by Mishels Uncertainty of Illness Scale (MUIS) [63], validated in a short form [64]. Translated into Danish during this study.
- *Negative beliefs about worry* is a validated subscale [65] of the MetaCognitions Questionnaire-30 [66] targeting metacognition. Translated into Danish in 2009 at Center for Psychiatric Research at Aarhus University Hospital.
- *Perceived risk of recurrence* is measured by a visual analogue scale from 1 to 100, as presented by Lebel et al. [27]. Translated into Danish during this study.

### Economic measures

For evaluating cost-effectiveness of TG-iConquerFear and for comparison of changes in health care usage between intervention arm and aTAU, information will be extracted from multiple Danish registries. Data will be obtained at end of study for the retrospective period from one year before intervention to one year after intervention, maximum 27 months.

- Health expenditure (register data from the Patient Registry (hospitalization and ambulatory visits), the Health Insurance Registry (Visits at primary care physician) and Drug Statistics Registry (Use of prescription drugs)
- Sickness absence (register hosted by the ministry of employment, (DREAM))

### Data management

The electronic data capture system REDCap will be used for data management with double data entry and subsequent shredding of paper questionnaires. During the study data will be processed and stored in pseudonym form in accordance with applicable legislation (EU GDPR and the Danish Data Protection Act), using REDCap and OPEN Analyse via OPEN Odense Patient data Explorative Network, Odense University Hospital, Region of Southern Denmark. When the study is finished, data will be transferred to the National Archives. Access to data is limited to listed authors by passwords.

### Statistical plan

#### Power calculation

The sample size is calculated for the primary outcome FCR measured by the FCRI. To detect a standardised mean difference in FCR (Cohen’s d = 0.5, group difference of 3.5 and a standard deviation of 7) with 90% power and two-sided alpha = 0.05 with two-sided t-test a sample size of 246 participants is desired. With a realistic dropout of 30%, 350 participants are required, and it seems realistic to recruit this among the estimated number of eligible participants. All invited CRC survivors with elevated FCR wishing to participate will be included in the study.

### Statistical analysis

A flowchart of participants and dropouts following the CONSORT guidelines will be drawn.

The statistical package STATA, latest version (StataCort, Texas, USA) will be used for all statistical analyses. All statistical tests will be two-sided (level of significance = 0.05). Where possible**χ**^2^-tests will be performed on all follow-up data to analyse proportions of participants above or below the clinical cut-off to calculate reliable and clinical change indices.

The influence of the intervention will be analysed for the primary and secondary outcomes. Linear mixed effects models will be used to analyse longitudinal differences between intervention and control groups. Association with functional disorders (health anxiety and bodily distress syndrome) and psychiatric disorders (anxiety and depression) will be evaluated with adjusted and unadjusted linear regressions analyses on baseline data.

### Dropout analysis

Available demographic data and psychosocial information will be compared and evaluated for participants and dropouts to assess generalizability. Any differences will be adjusted for in later analyses.

### Predictor analysis

The moderating effect of age, gender and cancer-related characteristics (e.g. stage of disease (I-IV), type of surgery, screening vs. non-screening) and actual situation (time since last visit at hospital, time until next visit, stoma) will be analysed using linear regression.

### Mediator analysis

The mediator’s uncertainty in illness, perceived risk of recurrence and negative beliefs about worry will be analysed by regression models with possible interaction with the intervention.

### Economic analysis

Multivariate analysis will be performed to explore any difference between the intervention and control group. The economic cost of the average participant in each group will be calculated based on register data.

### Dissemination

Results, whether positive, negative, or inconclusive, will be published in international, peer-reviewed journals reaching an appropriate audience (open access when possible). The RCT is registered at www.clinicaltrials.gov (NCT04287218) with a reference to results. Data will be presented at national and international congresses. An oral, public PhD defence will be held. If shown to be efficacious the results will be presented at cancer treating departments throughout Denmark to encourage implementation. Results will be published at the Danish Cancer Society’s webpage to inform future users. Study participants, if interested, will be emailed the results when the study is finished.

## Discussion

This RCT of TG-iConquerFear rigorously evaluates the efficacy of a therapeutic guided, Internet-delivered intervention for CRC survivors with clinical levels of FCR in a population based setting. After baseline assessment and semi-structured interview the participants are randomized to either the intervention group receiving Internet-delivered cognitive therapy or the augmented control group referred to a non-guided webpage. Follow-up measurements allow the detection of group differences in psychosocial aspects. Health service utilization and survival will also be investigated.

The developmental process of TG-iConquerFear has included CRC survivors, oncology nurses, psychiatrists, psychologists, the Patient’ and Relative’s Council at Lillebaelt Hospital and doctors working in the field of oncology. Internet delivery was chosen, as many CRC survivors are working, and traditionally men are more reluctant to participate in face-to-face therapy. We believe this combination of a careful development and a considerate delivery will make TG-iConquerFear successful in reducing FCR and has the potential to target a broader segment of the population than traditional face-to-face formats.

### Limitations/harms

Internet access is essential. The intervention cannot be delivered by paper, and CRC survivors without Internet are unable to participate. Internet-delivered therapy might not be sufficiently intensive to treat survivors with very high levels of FCR, and further treatment might be necessary.

We do not expect any harm done to the participants, but will ask participants to provide details of any adverse effect of the Internet intervention immediately post-treatment. If any participant shows signs of increasing distress, anxiety or depression during the intervention, the therapists are requested to address this.

## Conclusion

The results of this population based survey and RCT will contribute to the currently limited knowledge regarding psychosocial aspects of surviving CRC and how it can be addressed at a broad scale level. If proven effective, TG-iConquerFear can be implemented in the Danish healthcare system, and great effort will be made to encourage stakeholders like other cancer-treating clinical departments, general practitioners and the Danish Cancer Society to offer TG-iConquerFear to their survivors.

## Data Availability

The data generated during this study will be available from the corresponding author on reasonable request.

## Abbreviations

BDS: Bodily Distress Syndrome
CRC: Colorectal Cancer
DCCG: Danish Colorectal Cancer Group
FCR: Fear of Cancer Recurrence
FCRI-SF: Fear of Cancer Recurrence Inventory – Short Form
MCQ: Meta-Cognitions Questionnaire
MUIS: Mishels Uncertainty in Illness Scale
RCT: Randomized clinical trial
RKKP: The Danish Clinical Registries
SCL: Symptom CheckList
aTAU: Augmented Treatment as Usual
TG: Therapeutic Guided
VAS: Visual Analogue Scale
